# Nanocrystalline hydroxyapatite and zinc-doped hydroxyapatite as carrier material for controlled delivery of ciprofloxacin

**DOI:** 10.1007/s13205-011-0021-9

**Published:** 2011-08-24

**Authors:** G. Devanand Venkatasubbu, S. Ramasamy, V. Ramakrishnan, J. Kumar

**Affiliations:** 1 Crystal Growth Centre, Anna University, Chennai, 600025 Tamil Nadu India; 2 Department of Laser Studies, School of Physics, Madurai Kamaraj University, Madurai, Tamil Nadu India

**Keywords:** Biomaterials, Ciprofloxacin, Controlled release, Drug delivery, Hydroxyapatite, *Pseudomonas aeruginosa*, *Staphylococcus aureus*

## Abstract

In bone disorders infections are common. The concentration of majority of antibiotics is very low in the bone tissue. A high local dose can be obtained from the ciprofloxacin-loaded hydroxyapatite nanoparticles. The present study is aimed at developing the use of hydroxyapatite and zinc-doped hydroxyapatite nanoparticles as a carrier for ciprofloxacin drug delivery system. The ciprofloxacin-loaded hydroxyapatite and zinc-doped hydroxyapatite have a good antibacterial activity against *Pseudomonas aeruginosa* and *Staphylococcus aureus.* Hydroxyapatite and zinc-doped hydroxyapatite were prepared and characterized using X-ray diffraction, Transmission electron microscopy and inductively coupled plasma optical emission spectrometry. They were loaded with ciprofloxacin using optimized drug loading parameters. Drug loading, in vitro drug release and antimicrobial activity were analyzed. The influence of zinc on the controlled release of ciprofloxacin was analyzed. The results show that the presence of zinc increases the drug release percentage and that the drug was released in a controlled manner.

## Introduction

Nanotechnology deals with the manipulation of material characteristics (Ochekpe et al. 2009). Due to the large surface to volume ratio they are advantageous to attach drug molecules and other compounds (De Jong and Borm 2008). This is the main factor behind the application of nanotechnology in medical field for the delivery of drugs, proteins or genes. The drugs are effectively delivered by nanoparticles because nanoparticles are effectively taken up by the cells. They penetrate deep into tissue through fine capillaries and cross the fenestration present in the epithelial lining. Drug delivery can be either localized or targeted to a particular diseased site (Panyam and Labhasetwar 2003).

Controlled drug delivery is the technology by which the drug can be released at a predetermined rate for a long period of time into the blood or delivered at the target site (Szycher 1986). Unlike the traditional oral, intravenous drug delivery methods whereby the drug is distributed to both healthy and diseased tissue, in controlled local drug delivery high concentration of drug is achieved at the infected site. This leads to increase in therapeutic index and therapeutic efficacy and reduction in overall serum concentration and deleterious side effects to other organs (De Jong and Borm 2008; Melville et al. 2008; Noel MS et al. 2008; De Gaetano et al. 2005). Drug stability, optimized drug absorption, treatment continuation in natural phase and improvement in pharmacokinetic characteristics of drug can be achieved by localized drug delivery (Somberg 1989). In controlled drug delivery system the carriers play a vital role since they incorporate the drug, retain it and release it progressively with time. So properties such as drug incorporation and release; formulation stability and shelf life; biocompatibility, biodistribution, and functionality should be analyzed thoroughly when choosing a carrier for delivery of drugs. The drug release from any carrier depends upon solubility of drugs, microstructure of carrier, degradation of carrier and the bond between the drug and the carrier (De Jong and Borm 2008; Ginebra et al. 2006).

Bone diseases like osteomyelitis and osteoarticular infections which are associated with bacterial infection are highly complicated and involve operative debridement and removal of all foreign bodies followed by antibiotic therapy (Murugan and Ramakrishna 2006; Samit Kumar Nandi et al. 2009; Gautier et al. 2001). The blood circulation in these infected sites is limited and so the antibiotic distribution is also poor. So growth factors and antimicrobials should be supplied to the osseous site by site-specific drug delivery (Hae-Won Kim et al. 2004). By this method the drug action is localized and maintained for a long time. In spite of high drug concentration at the infection site the systemic side effects is low (Itokazu et al. 1999). By having a localized drug delivery we can maintain a high concentration of drug for a long time at the specific infected bone site by controlling the release of drug. The drug concentration can be controlled in a way that it neither reaches the toxic level nor falls below the minimum effective level (Ginebra et al. 2006). Even though high doses of antibiotics are administered by systemic routes, effective drug concentration is not achieved at the site of infection because of the impaired blood circulation, pharmacokinetic parameters of the drug and high elimination rate (Pham et al. 2002; Slosarczyk et al. 2000).

*Staphylococcus aureus* and *Pseudomonas aeruginosa* are the most important pathogens causing bone and joint infections, soft tissue and overwhelming sepsis. *Staphylococcus* is a Gram-positive and *Pseudomonas* is a Gram-negative bacterium. *S. aureus* expresses many surface adhesions that promote attachment to plasma and extra cellular matrix (ECM) proteins of the host cell. With the use of new and high-dose antibacterial agents the fear of new strain of *S. aureus* which is resistant to all available antibacterial agents arises. *S. aureus* can evade the immune response and antimicrobial therapy by another mechanism known as “Small colony variants” (SCVs), a sub population of *S. aureus* (Harris and Geoff Richards 2006; Uwe Joosten et al. 2005).

Ciprofloxacin (CPX), 1-cyclopropyl 6-fluro-1, 4-dihydro-4-oxo-7-(1-piperazinyl)-3-quinoline carbolitic acid is a broad-spectrum fluroquinolone antibacterial agent used in the treatment of both Gram-positive and Gram-negative microorganisms. It stops cell division by inhibiting DNA gyrase, a type II topoisomerase and topoisomerase IV. The minimum inhibition concentration (MIC) of CPX is 0.25–2 μg/ml for pathogens like *Staphylococcus aureus* and *Pseudomonas aeruginosa* which cause osteomyelitis. With such a low MIC the dosage of CPX is 700 mg twice a day for a period of 6 weeks to several months. The treatment is extremely long and in spite of that the percentage of cure is only 56%. This prolonged treatment and high dose of CPX leads to toxicity and development of resistant strains. So new sustained release systems have been proposed as an alternative for systemic treatment (Patel et al. 2006; Zeiler 1985; Murugan and Panduranga Rao 2002).

Polymethymethacrylate (PMMA) beads have been used initially to treat infectious bone diseases. But it has to be removed surgically as they are non-biodegradable. Resorbable biomaterials like collagen, fibrinogen and PLA when used do not replace bone grafting (Panyam and Labhasetwar 2003; Murugan and Ramakrishna 2006; Cheng and Kuhn 2007). Hydroxyapatite, a typical type of calcium phosphate bioceramic, is used as a carrier for delivery of drugs, non viral gene, antigen, enzyme and proteins because of their biocompatibility, bioactivity and high affinity towards drugs, proteins and DNA. Since Hydroxyapatite has a low solubility in physiological condition it can be used as a carrier for the local delivery of drugs both by surgical placement and injection. The controlled localized drug delivery from hydroxyapatite minimizes the toxicity to other organs by minimizing the drug concentration in the blood. It also avoids repeated dosage of drugs. Hydroxyapatite can bind to both positive and negative molecules by simple adsorption. So in the particulate form it is used for the delivery of various kinds of molecules like antibiotics, contraceptives, acetylsalicylic acid, hormones, insulin and anticancer drugs (Paul and Sharma 1999; Madhana Sundar et al. 2005; Szymura-Oleksiak et al. 2001). Hydroxyapatite is used in the controlled drug delivery for bone cancer, chronic osteomyelitis and in the delivery of agents with a low bone penetration and short biological half life. The concentration of drug depends upon the ability of the drug to penetrate through the micro pores of hydroxyapatite, rate of penetration and the pharmacokinetic profile of the drug. Since hydroxyapatite has a micro pore structure and excellent biological response to the physiological condition it can ensure slow release of the drug (Slosarczyk et al. 2000; Ingrid Russoni de Lima et al. 2006).

Zinc is an important trace element in all biological tissues. More than 300 types of enzymes like alkaline phosphates take part in bone metabolism. Zinc participates in the activity of these enzymes (Amit Bandyopadhyay et al. 2007). Zinc has a direct effect on the proliferative effect of osteoblastic cells and inhibitory effect on osteoclastic bone resorption (Tang et al. 2009). Besides these, zinc also takes part in nucleic acid metabolism, maintenance of membrane structure and its function, hormonal activity, biomineralization and pathological calcification (Mason 2006). It acts as an antioxidant. Many biochemical processes like carbohydrate metabolism, protein digestion and blood clotting require zinc. It plays an important role in immune system also. So it would be desirable to obtain hydroxyapatite with certain amount of zinc to enhance several biochemical processes (Say Chye Joachim Loo et al. 2008).

In the present work hydroxyapatite and zinc-doped hydroxyapatite were synthesized in nano form and characterized. Ciprofloxacin was loaded to both of these materials. The various parameters for drug loading were optimized. The drug loading percentage and drug release profile were analyzed and the influence of zinc on the release of ciprofloxacin discussed. The interaction between hydroxyapatite and drug was analyzed. Antimicrobial activity of drug loaded hydroxyapatite was analyzed against *Pseudomonas aeruginosa* and *Staphylococcus aureus.*

## Experimental procedure

### Synthesis and characterization of hydroxyapatite nanoparticles

Hydroxyapatite nanoparticles were synthesized by wet chemical precipitation reaction:

Aqueous suspension o f calcium hydroxide (Ca (OH)_2_) and orthophosphoric acid (H_3_PO_4_, 85%), both of analytical grade, were used as reagents for the preparation. One liter of an aqueous suspension of H_3_PO_4_ (0.6 M) was slowly added drop by drop to one litre of an aqueous suspension of Ca (OH)_2_ (1 M) while stirring for 2 h at room temperature (Pataquiva Mateus et al. 2007). Concentrated NaOH was added until a final pH of 11 was obtained. The white powder obtained was washed using deionized water and dried in an oven at 80 °C for 24 h (Edouard Jallot et al. 2005). Zinc-added hydroxyapatite was prepared by adding Zn(NO_3_)_2_.6H_2_O to the solution. Four samples of hydroxyapatite with zinc concentration of 2, 3, 4, 5 wt% (Gibson et al. 1999) were obtained.

Powder X-ray Diffraction (XRD, Seifert, JSO-DE BYEFLEX 2002, Germany) was utilized to identify the crystalline phase composition. The morphology and crystal structure of the product were observed by Transmission Electron Microscopy (TEM). The instrument was JEOL 2000Fx-II operated at 200 kV, High Resolution, analytical TEM with a W-source and a point–point resolution of 2 A˚. The functional groups present in the hydroxyapatite were analyzed by FTIR (FTIR, Perkin Elmer Spectrum One). Raman measurements were carried out using a Horiba Jobin–Yvon-HR 800 UV micro-Raman setup. The 325-nm line of He-Cd laser was used as the excitation source with a 2400 grooves mm^−1^ grating in the backscattering geometry. A 500-µm confocal pinhole was used to obtain high-resolution Raman spectra. Energy Dispersive X-Ray fluorescence (EDX) is done with Hitachi VP-SEM S-3400 N. Micro Hardness was analyzed using Leitz Wetzlar Miniload 2 hardness tester equipped with a Microduromat 4000 E fitted to the objective nosepiece (Reichert-Jung Ltd). X-ray Photo electron spectroscopy (XPS) was carried out by PHI 5000 Versa probe instrument. X-ray Fluorescence Spectroscopy (XRF) was measured using Rigaku ZSX Primus-II instrument.

### Drug loading

In order to load drug on hydroxyapatite nanoparticles, ciprofloxacin hydrochloride was dissolved in 100 ml of distilled water at different concentrations (5, 10, 20%). 1 g of hydroxyapatite nanoparticle was added to all the three drug solutions and stirred using magnetic stirrer for various time periods (20, 40, 80, 160 min) at various temperatures (40, 50, 60, 70 °C). Then the solution was left undisturbed overnight. The suspension was then centrifuged (224×*g*, 5 min) and the supernatant and precipitate were separated. The amount of drug loaded was determined by finding the difference in ciprofloxacin concentration in the aqueous solution before and after loading. Percentage of drug loading is calculated using the following equation:where *A* and *B* represent the initial and final drug concentration of the aqueous drug solution.

Drug loading on zinc-doped Hydroxyapatite was done in the same way where instead of hydroxyapatite zinc-doped hydroxyapatite was used. One gram of zinc-doped hydroxyapatite (2, 3, 4, 5 wt%) was added to 5% ciprofloxacin solution and stirred at optimized temperature and for optimized time.

Pellets of 100 mg, 8 mm in diameter pressed with 100 MPa of pure hydroxyapatite, zinc-added hydroxyapatite, ciprofloxacin-loaded hydroxyapatite and ciprofloxacin (control) were made and used in antimicrobial studies.

### Drug release-in vitro study

In order to determine the drug release profile, 100 mg of the drug-loaded hydroxyapatite and zinc-doped hydroxyapatite was introduced into a screw-capped glass bottle containing 50 ml of phosphate-buffered saline (PBS) medium at 37 °C and pH 7.4 under sterile condition. In vitro drug release study was done in static mode. The drug release study was done for a period of 600 h. 5 ml samples were withdrawn by a pipette and centrifuged at 224×*g* and replaced immediately with 5 ml of fresh PBS medium, which was accounted for when calculating the amount released. Ciprofloxacin concentration in the supernatant was measured spectrophotometrically at a wavelength of 274 nm. Drug release from zinc-doped hydroxyapatite samples was also done in the same way.

### Antibacterial activity

The antibacterial activity of pure hydroxyapatite, zinc-doped hydroxyapatite nanoparticles and ciprofloxacin-loaded hydroxyapatite and zinc-doped hydroxyapatite nanoparticles was tested by a quantitative diffusion disk test using *Staphylococcus aureus* and *Pseudomonas aeruginosa*. They were cultured on Luria–Bertani (LB) agar plates. Pellets of 100 mg, 8 mm in diameter pressed with 100 MPa of pure hydroxyapatite, zinc-doped hydroxyapatite, ciprofloxacin (control), ciprofloxacin-loaded hydroxyapatite and ciprofloxacin-loaded hydroxyapatite zinc-doped hydroxyapatite nanoparticles were aseptically placed at the center of the Petri plate. The plates were incubated for 24 h at 37 °C. The microbial inhibition zone was observed under optical microscope. The inhibition zone of the samples and control for the two different bacterial cultures were measured.

## Results and discussions

**Fig. 1:**
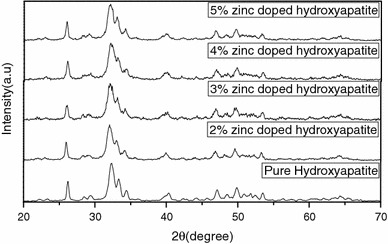
XRD image of pure hydroxyapatite and zinc-doped hydroxyapatite

Figure 1 shows the XRD pattern of pure hydroxyapatite and zinc-doped hydroxyapatite nanoparticles. It shows the formation of single-phase hydroxyapatite and the spectrum matches with the JCPDS values (09-0432). The major peaks indicate the crystalline form. The ‘*a*’ and ‘*c*’ value of hydroxyapatite nanoparticles are 9.416 and 6.886 Å, respectively, which are close to the lattice parameter of stoichiometric hydroxyapatite (Powder diffraction file ICDD 09-0432 *a* = 9.418 Å and *c* = 6.884 Å). The inter-planar distance *d* values agree well with the values estimated from TEM. Table 1 depicts these values. The crystalline size of hydroxyapatite nanoparticles can be calculated by Scherrer formula as follows:

**Table 1:** The comparison of values of *d* as calculated from the electron diffraction pattern and calculation from the XRD pattern

Plane (peak indexed as in XRD)	XRD *d* value Wave length λ = 1.542 Å	TEM d-value (first 3 rings) Camera constant = 0.0251 Å
002	3.48	3.44
211	2.79	2.77
212	2.25	2.24

where X_hkl_ is the crystallite size (nm), λ is the wavelength of monochromatic X-ray beam (nm) (λ = 0.15418 nm for CuKα radiation), β _½_ is the full width at half maximum for the diffraction peak under consideration (rad), ‘θ’ is the diffraction angle (°), and ‘K’ is a constant varying with crystal habit and chosen to be 0.9. In the XRD pattern, the (211) peak has the most distinct reflection. So, the mean crystalline size is calculated with the line broadening of the (211) reflection. The crystalline size of the pure hydroxyapatite crystals is about 52.00 nm.

In zinc-doped hydroxyapatite the zinc concentration [Zn/(Ca + Zn)] is expressed as the weight percentage of zinc. The peak in the XRD pattern of zinc-doped hydroxyapatite is identical to the XRD pattern of pure hydroxyapatite and no other crystalline phase is detected. As zinc concentration increases, the XRD peak of the samples become broader, indicating lower crystallinity due to the addition of zinc. Crystallinity, Xc is defined as the fraction of the cryslline phase in a sample volume. An empirical relation between Xc and β_1/2_ is commonly deduced according to the following equation:where *K*_A_ is a constant set at 0.24 and β_1/2_ is the FWHM of the (211) reflection (in degrees). (Table 2) shows the single crystal size and crystallinity of pure hydroxyapatite and Zinc doped hydroxyapatite. As the zinc content increased, both the crystallinity and crystal size decreased gradually. Similar observation has been made by Bonfield and his group for silicon substitution (de Araujo et al. 2007). The ionic radius is smaller for Zn^2+^ (0.074 nm) than that of Ca^2+^ (0.099 nm), which might have distorted the crystal structure of hydroxyapatite. The most important structure parameters are the lattice parameters and unit cell volume, which are shown in Table 3. The lattice parameter ‘*a*’ decreases with increasing Zn percentage. The lattice parameter ‘*c*’ also decreases with increasing Zn fraction. Such contraction in ‘*a*’ and ‘*c*’ due to the smaller ionic radius of Zn^2+^ (0.074 nm) compared with that of Ca^2+^ (0.099 nm) is reflected in the smaller unit cell volume with Zn^2+^ addition.

**Table 2:** Single crystal size and crystallinity of Zn-doped hydroxyapatite

Sample	Line width (211) FWHM (°)	Line width (211) FWHM (rad)	Average single crystal size D (Å)	Crystallinity (X_c_)
Hydroxyapatite	0.156	0.002722	52	3.641
2% Zinc-doped hydroxyapatite	0.161	0.00280945	50	3.312
3% Zinc-doped hydroxyapatite	0.267	0.00465915	30	0.726
4% Zinc-doped hydroxyapatite	0.292	0.0050954	27	0.555
5% Zinc-doped hydroxyapatite	0.347	0.00605515	23	0.330

**Table 3:** Cell parameters of zinc-doped hydroxyapatite

Sample	*a* (Å)	*c* (Å)	*V* (Å)
Hydroxyapatite	9.4360 ± 0.0818	6.8928 ± 0.1273	531.500
2% Zinc-doped hydroxyapatite	9.4255 ± 0.0582	6.8860 ± 0.1069	530.562
3% Zinc-doped hydroxyapatite	9.3791 ± 0.0949	6.8590 ± 0.0152	522.531
4% Zinc-doped hydroxyapatite	9.3516 ± 0.0022	6.8303 ± 0.0022	522.030
5% Zinc-doped hydroxyapatite	9.3365 ± 0.0163	6.8275 ± 0.1656	521.211

**Fig. 2:**
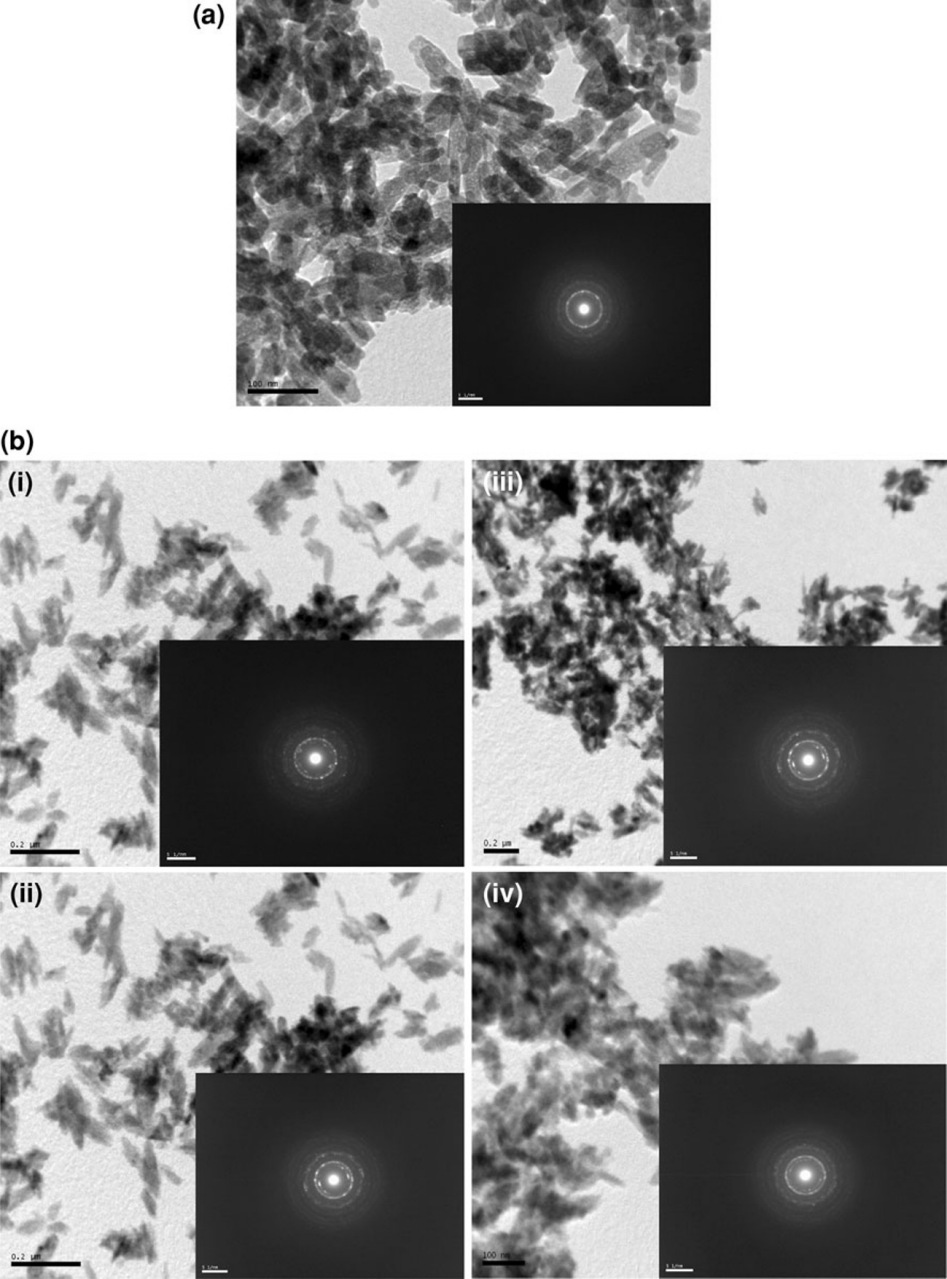
**a** Bright field TEM image of pure hydroxyapatite. **b** TEM image of *i* 2 wt% zinc-doped hydroxyapatite, *ii* 3 wt% zinc-doped hydroxyapatite, *iii* 4 wt% zinc-doped hydroxyapatite, *iv* 5 wt% zinc-doped hydroxyapatite

The TEM image of pure hydroxyapatite nanoparticle is presented in Fig. 2a. The picture confirms the formation of nanocrystalline powder. The particles are uniform and their sizes range from 10 to 20 nm due to agglomeration. They show needle-like structure. Fine discrete particles of nano range are clearly visible in the loosely agglomerated powder. The hydroxyapatite nanoparticles are crystalline as can be seen from XRD. The nanocrystals exhibit sharp edges and corners. The rate of nucleus formation and the rate of growth are related to the concentration of reactants, super saturation, temperature, stirring rate etc. TEM micrographs of different zinc concentration are shown in Fig. 2bi, ii, iii, iv. TEM micrographs show the influence of zinc addition. On zinc incorporation the morphology of hydroxyapatite changes. As the concentration of zinc increases, agglomeration of particles also increases. The zinc addition in hydroxyapatite structure causes a decrease in the hydroxyapatite crystalline nature which may be due to both substitutional and interstitial site occupancy of zinc as discussed in the interpretation of Raman spectra and XRD. There are no additional selected area diffraction rings with addition of zinc up to 5 wt%. This confirms the structure as seen in XRD. The *d*-values as calculated from the TEM electron diffraction rings exactly match with the prominent peaks obtained from the XRD analysis (Fig. 1).

**Fig. 3:**
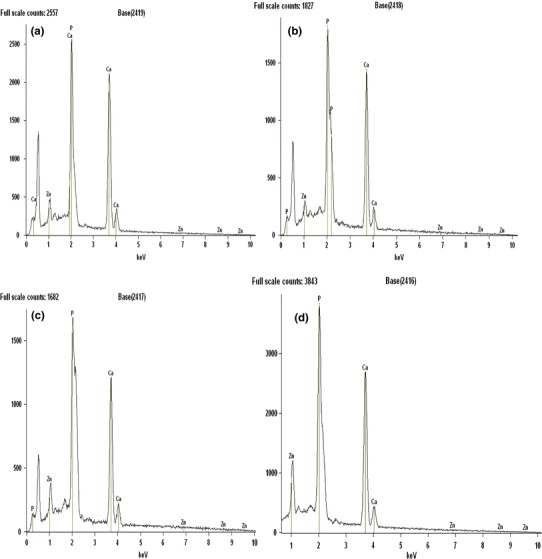
EDX spectrum of zinc-doped hydroxyapatite. **a** 2% Zinc-doped hydroxyapatite, **b** 3% zinc-doped hydroxyapatite, **c** 4% zinc-doped hydroxyapatite, **d** 5% zinc-doped hydroxyapatite

EDX analysis of Zinc-doped hydroxyapatite is given in Fig. 3. It clearly showed the presence of Ca, P, Zn and O in the zinc-doped hydroxyapatite structure. The decrease in the Ca/*P* upon increase in the zinc concentration suggests that Ca^2+^ in crystal lattice of hydroxyapatite reduces with the increase of Zn^2+^. The expected [Ca]/[P] wt ratio is 2.15, according to the stoichiometry of pure hydroxyapatite. Table 4 indicates that the stoichiometry deviation of the ([Ca] + [Zn])/[P] wt ratios are higher than the [Ca]/[P] ratios of the pure samples. In the doped samples, apart from the incorporation of carbonate groups, other mechanism can cause deviation from stoichiometry. In all the samples the [Ca]/[P] wt ratios are higher than the expected ones and they are around 2.4. It means that the samples are about 14% P deficient, but these values are within the [Ca]/[P] limits that the hydroxyapatite structure can accommodate. They are also compatible with the values found in the mineral part of human bones. EDX measurements indicated that the average [Ca]/[P] ratios in the pure sample is 2.83, 30% higher than the expected values of 2.15. This difference can be explained by incorporation of carbonate groups substituting the phosphate ones in the hydroxyapatite matrix. This is an advantage of the chemical route used in the present work since the carbonate groups are known to accelerate the re-absorption of hydroxyapatite implants. In the Zn^2+^-doped samples, the EDX results indicates the deviation from stoichiometry, with ([Ca] + [Zn])/[P] ratios of about 2.24, 14% higher than the 2.15 expected value. This indicates that the dopant enhances the incorporation of carbonate groups. Synthetic hydroxyapatite is similar to the hydroxyapatite of human bones and tooth and contains carbonate groups. The carbonate groups occupy phosphate sites leading to P deficiency. The pure hydroxyapatite samples were prepared in air and thus there is a reasonable chance that carbonates are formed via reaction of the hydroxyl groups in the aqueous suspension with the CO_2_ present in the atmosphere forming H_2_CO_3_ that is easily dissolved in water. The carbonate groups are then free to be incorporated in the hydroxyapatite nanoparticles occupying the phosphate sites in the hydroxyapatite structure. This substitution, however, did not cause appreciable change in the XRD pattern of hydroxyapatite since the X-Ray scattering factors for phosphates and carbonates are not very different and the amount of phosphate substitution by carbonates is quite low: around 30% and 14% in Zn-doped hydroxyapatite according to the EDX results, below the detection limit of XRD in this particular case. On the other hand, the reabsorption of implants made with hydroxyapatite is faster when carbonate groups are present in the hydroxyapatite structure (Koort et al. 2008).

**Table 4:** Calculation of weight concentration ratios of hydroxyapatite and zinc-doped hydroxyapatite via EDX

Sample	Ca/P (wt%)	(Ca + Zn)/P (wt%)
Hydroxyapatite	2.79	2.79
2% Zinc-doped hydroxyapatite	2.60	2.62
3% Zinc-doped hydroxyapatite	2.56	2.50
4% Zinc-doped hydroxyapatite	2.27	2.31
5% Zinc-doped hydroxyapatite	2.20	2.24

The zinc-doped hydroxyapatite samples were analyzed by ICP-OES spectroscopy to find out the amount of zinc present in the zinc-doped hydroxyapatite samples. Table 5 gives the amount of zinc in the zinc-doped hydroxyapatite samples. The amount of zinc increases with increase in the amount of zinc added in the mother liquid.

**Table 5:** ICP-OES values of weight percentage of zinc in zinc-doped hydroxyapatite

S. No	Sample	Zinc concentration (Weight %)
1	2% Zinc-doped Hap	0.4990
2	3% Zinc-doped Hap	0.7400
3	4% Zinc-doped Hap	0.9710
4	5% Zinc-doped Hap	1.3740

The amount of zinc in the zinc-doped hydroxyapatite was quantitatively confirmed by XRF. The relative amounts of Ca, Zn and P in the nanoparticles are listed in Table 6. The XRF results agree with the ICP-OES results regarding the zinc concentration. The zinc content of the samples is lower than those of corresponding amount of starting material. This implies that some of the Zinc ions remain in the mother solution after precipitation. Pure hydroxyapatite has a theoretical composition of Ca/P wt ratio, 2.15. The Ca/P wt ratios of pure and all Zinc-doped hydroxyapatite powders were higher than that of the stoichiometric hydroxyapatite. The addition of Zinc seems to affect the stoichiometry of hydroxyapatite. The stoichiometry of Zinc-doped hydroxyapatite is compared with the (Ca + Zn): P ratio because Zinc is likely to replace some Ca ions. The increase in the amount of Zinc decreased the (Ca + Zn): P ratio. This might be due to the generation of crystal defects from Zinc substitution. The (Ca + Zn): P ratio for 2 and 3% zinc-doped hydroxyapatite is 2.67 and 2.64, respectively. The Ca/P ratio for pure hydroxyapatite is 2.83. The (Ca + Zn): P ratio for 2 and 3% zinc-doped hydroxyapatite shows a slight variation from the Ca/P ratio of pure hydroxyapatite. For 4 and 5% Zinc-doped hydroxyapatite the (Ca + Zn): P ratio is 2.35 and 2.29, respectively, where the variation is larger. This shows that in 2 and 3% zinc-doped hydroxyapatite most of the Zn^2+^ ions substitute the position of Ca^2+^ in the apatite lattice. However, in 4 and 5% zinc-doped hydroxyapatite it seems that not all the Zn^2+^ ions are in the substitutional position. This is confirmed from the XRD, FTIR and EDX analysis where there is no existence of any noticeable diffraction peak and band other than that of hydroxyapatite.

**Table 6:** Chemical composition of hydroxyapatite and zinc-doped hydroxyapatite by XRF

Sample	Ca (wt%)	Zn (wt%)	P (wt%)	Zn: (Ca + Zn) (wt%)	Ca: P (wt%)	(Ca + Zn): P (wt%)	Ca: (P + Zn) (wt%)	Zn: (P + Zn) (wt%)
Hydroxyapatite	73.940	0	26.060	0	2.83	2.83	2.83	0
2% Zinc-doped hydroxyapatite	72.230	0.533	27.237	0.00732	2.65	2.67	2.60	0.019
3% Zinc-doped hydroxyapatite	71.919	0.611	27.470	0.00842	2.61	2.64	2.56	0.021
4% Zinc-doped hydroxyapatite	69.229	0.921	29.850	0.01312	2.31	2.35	2.24	0.029
5% Zinc-doped hydroxyapatite	68.370	1.300	30.330	0.01865	2.25	2.29	2.16	0.041

**Fig. 4:**
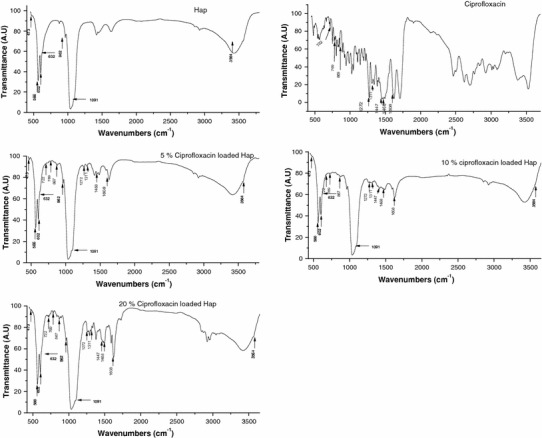
FTIR spectrum of ciprofloxacin-loaded hydroxyapatite

The structure of the ciprofloxacin-loaded hydroxyapatite was analyzed using FTIR spectroscopy As Shown in (Fig. 4). Characteristic structural bands of both hydroxyapatite and ciprofloxacin were observed for all hydroxyapatite/drug samples. The hydroxyapatite loaded with drug represents mixed bands typical of hydroxyapatite (P–O at 566, 602, 962, 1,091 and O–H at 632, 3,564) and ciprofloxacin(C=C stretching at 1,608, CH_2_ bending at 1,468, mixed vibrations at 1,311, CH in plane bending at 1,272, CN stretching at 867). The corresponding band intensities increase with increase in the drug loading percentage. The drug-free hydroxyapatite shows little difference confirming that the drug has no significant effect on the structure.

**Fig. 5:**
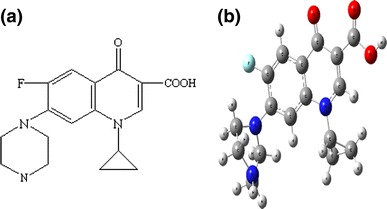
**a** Structure of ciprofloxacin, **b** optimized geometry of ciprofloxacin deduced through Gaussian 03 W

**Fig. 6:**
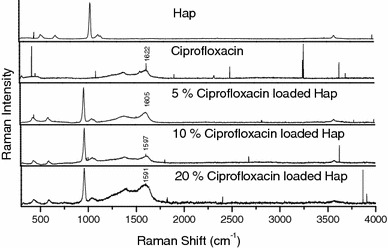
Raman spectrum of ciprofloxacin-loaded hydroxyapatite

The structure of ciprofloxacin and the optimized geometry deduced through Gaussian 03 W is given in (Fig. 5). The Raman spectrum of ciprofloxacin-loaded hydroxyapatite is shown in (Fig. 6). The observed Raman frequencies agree quite well with the literature. The most prominent peaks at 1,379 and 1,622 are associated with the ring vibrations. The Raman spectrum of hydroxyapatite loaded with various concentration of drug is given in (Fig. 6). It is interesting to note that the above peaks are more pronounced and it is highest for 5% and least for 20% drug loading. The frequencies of vibrations of PO_4_^3−^ ion are also shifted to lower wave number region. The ring stretching vibration at 1,622 cm^−1^ for drug gets shifted to lower wave number region on coating of the drug on hydroxyapatite. It shifts to 1,605 cm^−1^ for 5% drug concentration, 1,597 cm^−1^ for 10% drug concentration and 1,591 cm^−1^ for 20% drug concentration. The shift is more for 20% while considering the structure of the drug there are three possible sites for molecular adsorption on hydroxyapatite: (1) the aromatic ring (2) the carbonyl groups and (3) lone pair electrons of the hydrogen atoms.

According to the charge transfer idea of SERS, the charge transfer between the adsorbed molecule and the substrate is controlled by molecular frame vibration. It is modulated by the vibration of those adsorbed groups that are linked directly to the surface. A perpendicular orientation of the drug molecule would result through carbonyl groups. In the present case the carbonyl stretching mode is not observed or may be very week. This eliminates the possible adsorption through carbonyl groups. The observation of enhancement in the intensity of ring vibrations suggests that there will be charge transfer between the drug and the hydroxyapatite through π bonds. The observation of shifts in the frequencies of PO_4_ vibrations suggests that the drug molecules are very nearer to the PO_4_ groups. Hence it can be concluded that the drug molecule has an interaction with hydroxyapatite with a flat on orientation with their π electron clouds.

### Drug loading

Drug loading efficiency of hydroxyapatite was examined as described previously. It is dependent on the concentration of drug and the ratio of drug and hydroxyapatite. The drug loading percentage increases with increase in these parameters. It increases and becomes constant at a particular level. The variation in drug loading with change in drug concentration is given in (Table 7). For 5% drug concentration the drug loading is 90% and it increases to 96% at 10% drug concentration and remains same for 20% drug concentration. The variation in drug loading with change in temperature is given in (Table 8). With 5% drug concentration and 40-min stirring time, as the temperature is increased the drug loading increases and attains a maximum of 90% at 60 °C and then it becomes constant. With 5% drug concentration and temperature 60 °C the variation in drug loading with increase in stirring time is given in (Table 9). Maximum drug loading is obtained in 40 min and then it becomes constant. The optimized parameters, 40-min stirring time and 60 °C, are used for drug loading.

**Table 7:** Drug loading percentage with various drug concentrations

S. No	Drug concentration (%)	Drug loading (%)
1	5	90
2	10	96
3	20	96

**Table 8:** Drug loading percentage with different temperature

S. No	Temperature ( °C)	Drug loading (%)
1	40	85
2	50	87
3	60	90
4	70	90

**Table 9:** Drug loading percentage with different stirring time

S. No	Time (min)	Drug loading (%)
1	20	87
2	40	90
3	80	90
4	160	90

Ciprofloxacin (5% concentration) was loaded on all the four zinc-doped hydroxyapatite samples. The drug loading with various zinc concentration is given in (Table 10). There is no change in the drug loading percentage with the incorporation of zinc.

**Table 10:** Drug loading percentage with different zinc concentration

S. No	Zinc concentration (%)	Drug loading (%)
1	2	90
2	3	90
3	4	90
4	5	90

### In vitro drug release

Many carrier systems are used for the controlled delivery of ciprofloxacin. Poly (dl)-lactide matrix are used for infection therapy (Chouhan and Bajpai 2010). It showed a controlled release in in vivo analysis. Ciprofloxacin-loaded poly (2-hydroxyethyl methacrylate) shows a biphasic release curve. It proved moderately effective in killing both gram-positive and gram-negative bacteria (Norris et al. 2005). Ultrasonically controlled release of ciprofloxacin is also achieved by pHEMA hydrogels (Chouhan and Bajpal 2009). Poly-(2-hydroxyethyl methacrylate) is used for the delivery of 5-fluro-uracil (Vijayakumar and Jain 2007). Ciprofloxacin-loaded Human serum Albumin nanoparticles give a controlled release of 20 h (Soriano de Souza et al. 2011). Since hydroxyapatite nanoparticles are naturally present in bone, they are used for the delivery of antimicrobial like chlorhexidine and gentamicin for bone infections (Liu et al. 2010; Akashi et al. 2001).

**Fig. 7:**
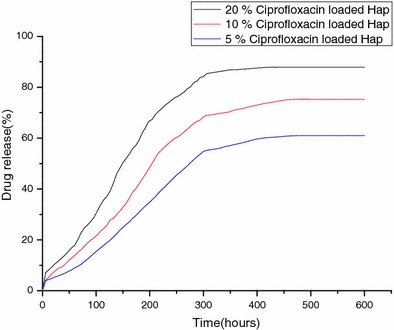
Drug release percentage from hydroxyapatite

Ciprofloxacin was loaded on hydroxyapatite to investigate the efficacy of the drug delivery system. The different drug release profiles were well illustrated in (Fig. 7). The estimated percentages of drug released in 600 h were found to be 88, 75 and 61% from 20, 10 and 5% drug-loaded samples, respectively. The drug is released gradually over a period of time. This shows that the drug is released in a controlled manner. The amount of drug released in 24 h of time is well above the minimum inhibition concentration of CPX which is 0.25–2 μg/ml for pathogens like *S. aureus* and *P. aeruginosa* which cause bone infections. Such a release profile observed could provide a rapid delivery of drug to give antibacterial effects at the infected site and a sustained release to aid long-term healing and avoid the toxic and adverse systemic effects caused by high concentration of antibiotics. The slow release of ciprofloxacin is due to the presence of trapped carboxylic group, which reacts with Ca in the hydroxyapatite and zinc-doped hydroxyapatite (Baker 1987). The plotted graphs revealed that all the samples showed typical drug release pattern with a lag time which was similar to the pattern for the membrane diffusion process of the drug (Matsunaga et al. 2010). The release of ciprofloxacin from hydroxyapatite nanoparticles exhibits a typical two-stage release mechanism. The drug release is high during the initial time and then it reduces and becomes constant. Ciprofloxacin release during the initial burst stage is due to desorption of ciprofloxacin molecules that are located on the surface of the particles. These particles do not strongly interact with the hydroxyapatite nanoparticles. During the in vitro drug release analysis the hydroxyapatite absorbs the surrounding fluid into the nanoparticles. This leads to the dissolution and exclusion of the loaded ciprofloxacin. When the large particles break into smaller particles more ciprofloxacin gets exposed to the fluid. When the loosely adsorbed ciprofloxacin had almost completely desorbed, the drug release becomes slow. The slow release of ciprofloxacin molecules is due to the incorporation of the drug hydroxyapatite nanoparticles. This results from the dissolution of the hydroxyapatite nanoparticles.

**Fig. 8:**
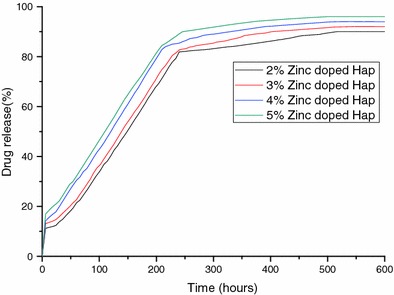
Drug release percentage from the zinc-doped hydroxyapatite

Drug release profile from the zinc-doped hydroxyapatite is given in (Fig. 8). The percentage of drug released increases with increase in the zinc concentration. The percentages of drugs released in 600 h were estimated to be 85, 88, 92 and 15.60% from 2, 3, 4, 5% zinc-doped samples respectively. There is a sudden outburst of drug in the initial few hours and then the drug is released in a controlled manner. The drug release percentage increases with increase in the zinc percentage from 2 to 5%. The concentration of zinc increases in the doped sample which is given by the ICP analysis. But there is a slightly higher increase in the drug release from 4 and 5% zinc-doped hydroxyapatite. The release of zinc from zinc-doped hydroxyapatite along with ciprofloxacin release may be the reason for the increase in drug release. The mechanism by which zinc release induces higher drug release is not known.

The incorporation of zinc into hydroxyapatite and the mechanism of zinc incorporation have been studied extensively by various researchers (Tang et al. 2009; Sogo et al. 2004). The release of zinc from zinc-doped hydroxyapatite and the increase in osteoblastic cell growth by zinc-doped hydroxyapatite are well documented (Ito et al. 2002; Vojislav Stanic et al. 2010). This simultaneous release of zinc and ciprofloxacin will promote both osteoblastic cell growth and antimicrobial activity.

### Antibacterial activity

**Fig. 9:**
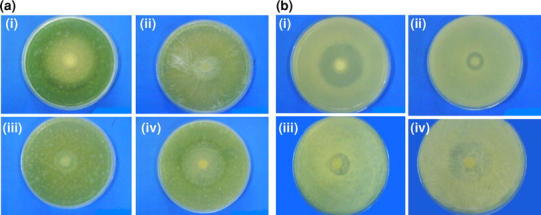
**a** Antimicrobial activity of *i* Ciprofloxacin (control), *ii* 5% ciprofloxacin-loaded hydroxyapatite, *iii* 10% ciprofloxacin-loaded hydroxyapatite, *iv* 20% ciprofloxacin-loaded hydroxyapatite against *Pseudomonas aeruginosa.***b** Antimicrobial activity of *i* Ciprofloxacin (control), *ii* 5% ciprofloxacin-loaded hydroxyapatite, *iii* 10% ciprofloxacin-loaded hydroxyapatite, *iv* 20% ciprofloxacin-loaded hydroxyapatite against *Staphylococcus aureus*

**Fig. 10:**
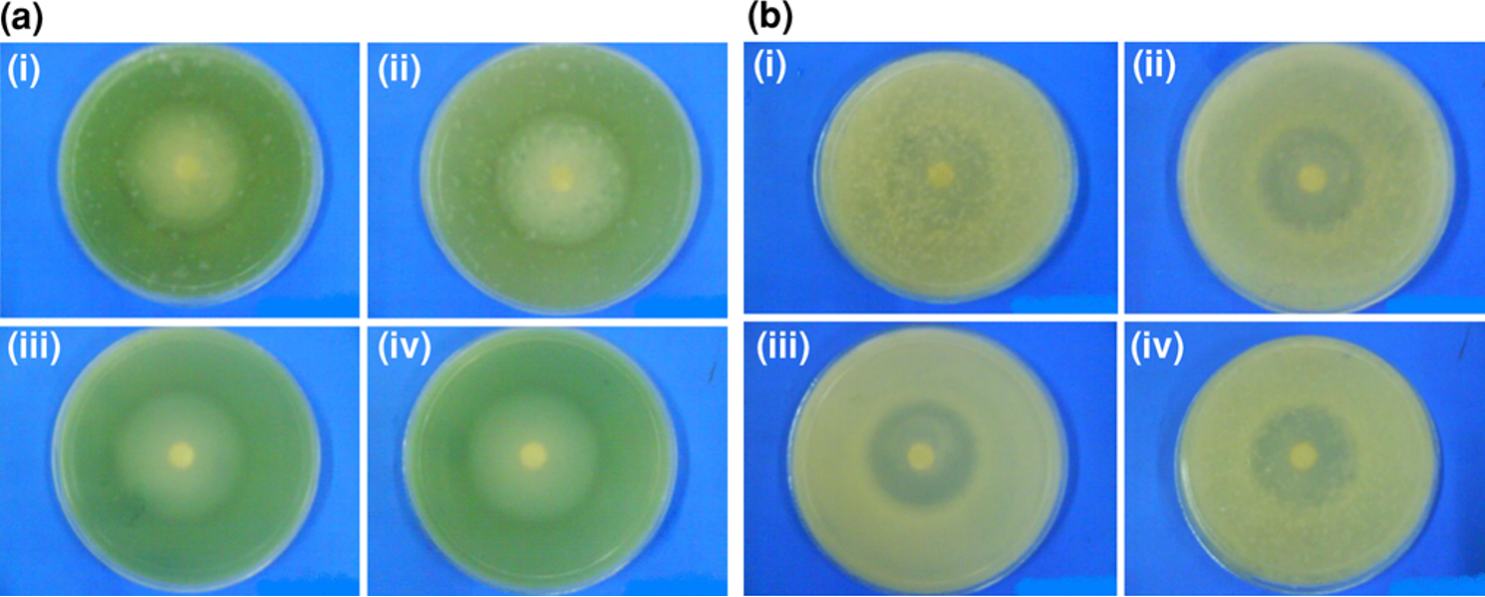
**a** Antimicrobial activity of *i* ciprofloxacin-loaded 2 wt% zinc-doped hydroxyapatite, *ii* ciprofloxacin-loaded 3 wt% zinc-doped hydroxyapatite, *iii* ciprofloxacin-loaded 4 wt% zinc-doped hydroxyapatite, *iv* ciprofloxacin-loaded 5 wt% zinc-doped hydroxyapatite against *Pseudomonas aeruginosa*. **b** Antimicrobial activity of *i* ciprofloxacin-loaded 2 wt% zinc doped hydroxyapatite, ii ciprofloxacin loaded 3 wt% zinc doped-hydroxyapatite, *iii* ciprofloxacin loaded 4 wt% zinc doped hydroxyapatite, *iv* ciprofloxacin-loaded 5 wt% zinc-doped hydroxyapatite against *Staphylococcus aureus*

The antibacterial activity of ciprofloxacin-loaded hydroxyapatite and zinc-doped hydroxyapatite against different micro-organism was monitored. Disk diffusion method was used to determine the zone of inhibition. The results of antibacterial activity of ciprofloxacin-loaded hydroxyapatite and zinc-doped hydroxyapatite are given in (Table 11). The zone of inhibition around the ciprofloxacin (control) and ciprofloxacin-loaded hydroxyapatite, against *Pseudomonas aeruginosa* is given in Fig. 9a and against *Staphylococcus aureus* in Fig. 9b, respectively. The zone of inhibition around the ciprofloxacin-loaded zinc-doped hydroxyapatite against *Pseudomonas aeruginosa* is given in Fig. 10a and against *Staphylococcus aureus* in Fig. 10b. The zone of inhibition increases with increase in the ciprofloxacin concentration. This is relevant with the apparent amount of drug release. When the amount of drug release increases the zone of inhibition also increases. All the ciprofloxacin-loaded zinc-doped hydroxyapatite nanoparticles have the greater antibacterial activity than ciprofloxacin-loaded pure hydroxyapatite, and the ciprofloxacin-loaded antibacterial activity of zinc-doped hydroxyapatite gets higher with the increase of zinc concentration. Since the crystallinity of hydroxyapatite decreases with increase in wt% of Zn^2+^, the surface area of the nano grains increases thereby forming bonds with the microorganisms and causes cell death. The proposed antibacterial mechanism of Zn^2+^ is that the ions in the crystal surface form strong bonds with thiole, imidazole, amino and carboxyl groups of microorganism membrane proteins, causing structural changes. A microorganism membrane with structural changes exhibits a significant increase in permeability, leaving the microorganism cells incapable of properly regulating transport through the plasma membrane and, finally, causing cell death (Vojislav Stanic et al. 2010).

**Table 11:** Zone of inhibition of as-synthesized ciprofloxacin-loaded hydroxyapatite and ciprofloxacin-loaded hydroxyapatite zinc-doped hydroxyapatite

S. No	Sample	Zone of Inhibition (cm)	
		*Staphylococcus aureus*	*Pseudomonas aeruginosa*
1	Ciprofloxacin (control)	2.0	2.4
2	5% Ciprofloxacin-loaded hydroxyapatite	1.7	2.15
3	10% Ciprofloxacin-loaded hydroxyapatite	2.0	2.6
4	20% Ciprofloxacin-loaded hydroxyapatite	3.0	4.1
5	2 wt% Zinc-doped hydroxyapatite + 5% ciprofloxacin	1.8	2.0
6	3 wt% Zinc-doped hydroxyapatite + 5% ciprofloxacin	1.8	2.0
7	4 wt% Zinc-doped hydroxyapatite + 5% ciprofloxacin	1.9	2.1
8	5 wt% Zinc-doped hydroxyapatite + 5% ciprofloxacin	1.9	2.1

## Conclusion

A drug delivery system with hydroxyapatite and zinc-doped hydroxyapatite was developed for use in bone infections. Both pure hydroxyapatite and zinc-doped hydroxyapatite can be used as drug delivery system for the controlled delivery of ciprofloxacin. Ciprofloxacin can be loaded on hydroxyapatite to enhance wound healing as a drug delivery system. Ciprofloxacin-loaded zinc-doped hydroxyapatite can be used for drug delivery and induce cell growth. The antimicrobial property increases with increase in the drug concentration and the amount of zinc. The drug loading depends on the concentration of drug, temperature and stirring time. The drug loading percentage increases with increase in drug concentration, temperature and stirring time and then becomes constant. The drug release from zinc-doped hydroxyapatite is higher than the drug release from pure hydroxyapatite. The drug release profile from both pure and zinc-doped hydroxyapatite shows a controlled release. The higher release of ciprofloxacin from zinc-doped hydroxyapatite is due to the release of zinc from zinc-doped hydroxyapatite. Both zinc and ciprofloxacin will be released when drug is loaded on zinc-doped hydroxyapatite. This will induce both cell growth and antimicrobial activity.
